# Knowledge, attitude and practices about rabies management among human and animal health professionals in Mbale District, Uganda

**DOI:** 10.1186/s42522-020-00031-6

**Published:** 2020-12-14

**Authors:** Fred Monje, Joseph Erume, Frank N. Mwiine, Herbert Kazoora, Samuel George Okech

**Affiliations:** 1 Ministry of Agriculture, Animal Industry and Fisheries, P.O. Box 102, Entebbe, Uganda; 2 College of Veterinary Medicine, Animal Resources and Biosecurity (COVAB), Makerere University, P.O. Box 7062, Kampala, Uganda; 3 African Field Epidemiology Network (AFENET), P.O Box 12874, Kampala, Uganda

**Keywords:** Rabies, Knowledge, Attitude, Professionals, Human-health, Animal-health, Zoonosis, One health, Uganda

## Abstract

**Objective:**

To assess the knowledge, attitude and practices (KAP) of animal and human health professionals towards rabies management and also to establish the level of relationship between KAP.

**Methods:**

A cross-sectional study was conducted between December 2012 and March 2013 among 147 randomly selected animal and human health professionals in Mbale District. Of these, only 16 were animal health professionals. Quantitative data was obtained using a semi-structured questionnaire while qualitative data was obtained from 4 Focus Group Discussions (FGDs) and 2 Key Informant (KI) interviews. Quantitative data was entered into EpiInfo version 3.5.1 and proportions computed while qualitative data was summarised into themes and sub-themes resulting from content analysis of interview scripts.

**Findings:**

Of all the respondents, only 44% (65/147) had sufficient knowledge about rabies while 25% (37/147) had positive attitude towards rabies management. A half of the respondents (50%, 73/147) had limited good practices. Respondents knowledgeable about rabies were more likely to have positive attitude towards rabies management (OR = 3.65; 95% CI: 1.60–8.3) while respondents with positive attitudes, were more likely to have good practices towards rabies management (OR: 2.22; 95% CI: 1.01–4.86).

**Conclusion:**

Respondents had low knowledge, negative attitude and limited good practices of rabies management. Regular refresher trainings about rabies to broaden staff knowledge and improve their attitudes and hence practices of rabies management should be conducted by the District leaders. Harnessing multi-sectoral and multi-disciplinary collaborative efforts (“One Health” approach) for rabies control should be instituted to reduce the incidence of the disease in the District.

**Supplementary Information:**

The online version contains supplementary material available at 10.1186/s42522-020-00031-6.

## Background

Rabies is a major zoonotic disease threatening global public health [1] and causes about 55,000 human deaths annually [2] with Asia being the worst affected, followed by Africa [3–5]. Though it is a global concern, rabies is frequently under-reported in developing countries [6] and has therefore become an important neglected disease [7]. The probable reasons for rabies being neglected is under-reporting and the nature of deaths that are scattered and never match the kind of crises that get other infectious and non-infectious disease epidemics the comparatively high level of national and global attention [8]. Consequently, the disease has remained uncontrolled in developing countries despite the existence of safe, cheap and effective vaccines for dogs [1].

Among emerging factors attributed to the failure in control and prevention of rabies are poor knowledge, attitudes and practices regarding rabies among stakeholders as has been documented in various countries [1, 9, 10] . In Africa, studies in Tanzania revealed that veterinary field staff and medical practitioners had low awareness and poor knowledge of zoonoses, rabies inclusive [11]. Concerted studies showed that knowledge and practices of practising doctors towards anti-rabies prophylaxis was highly wanting [12, 13] especially the management of suspected rabies cases in humans. Moreover, studies conducted in Tanzania indicated that increased awareness of diseases among health workers and the community was the most important factor in the control of diseases [14]. In Uganda, animal bites in humans were recorded as 15,320 bites in 2007; 16,834 bites in 2008; 17,399 bites in 2009; 11,173 in 2010 and 12,718 in 2011 [15]. In the Eastern Region of Uganda, Mbale District recorded the highest number of bites compared to other districts. While in 2009 Mbale recorded 882, the second highest recorded 487 (Kumi District) and the lowest recorded 16 (Budaka district). The highest, second highest and lowest numbers for 2010 and 2011 respectively are: 432 (Mbale District), 259 (Bukwo District), 0 (Budaka District) and 251 (Mbale), 240 (Kaabong District), 4 (Mayuge District). Comparative figures were not available for 2007 and 2008 in which Mbale reported 802 and 625 bites respectively. The available reports indicate up to 93 human deaths in 2010 alone due to suspected rabies. These cases of human deaths were clinically diagnosed [15]. The established norm in Mbale District is that both animal and human health professionals are expected to play key roles in the control of rabies. Animal health professionals spearhead anti-rabies vaccinations of pets and community sensitisation in an attemp to control rabies arising from domestic animal bites/scratches. Similarly, the human health professionals provide anti-rabies vaccines to human victims bitten or scratched by either animals with unknown vaccination record or suspected to be rabid and also conduct community education on the control of rabies [15]. Despite these efforts, Mbale District persistently had the highest number of animal bites (a proxy to rabies) in Eastern Uganda – averaging 587 animal bites in humans for five consecutive years (2007–2011) [15]. This poses a huge threat to human life since animal bites are closely linked to Rabies, a highly fatal disease. This is supported by a study which found out that almost all human rabies cases are related directly to animal bites hence, primary disease prevention necessitates reduction of suspected exposures [16]. It is unclear why animal bites have consistently remained high in Mbale District despite the control measures. Gaps in Knowledge, Attitudes and Practices (KAP) among animal and human health professionals may present a possible explanation to the above scenario. Thus, this study sought to establish the level of KAP and relationship between them (knowledge and attitudes; knowledge and practices; attitudes and practices) among animal and human health professionals towards rabies management.

## Methods

### Study area and study population

**Fig. 1:**
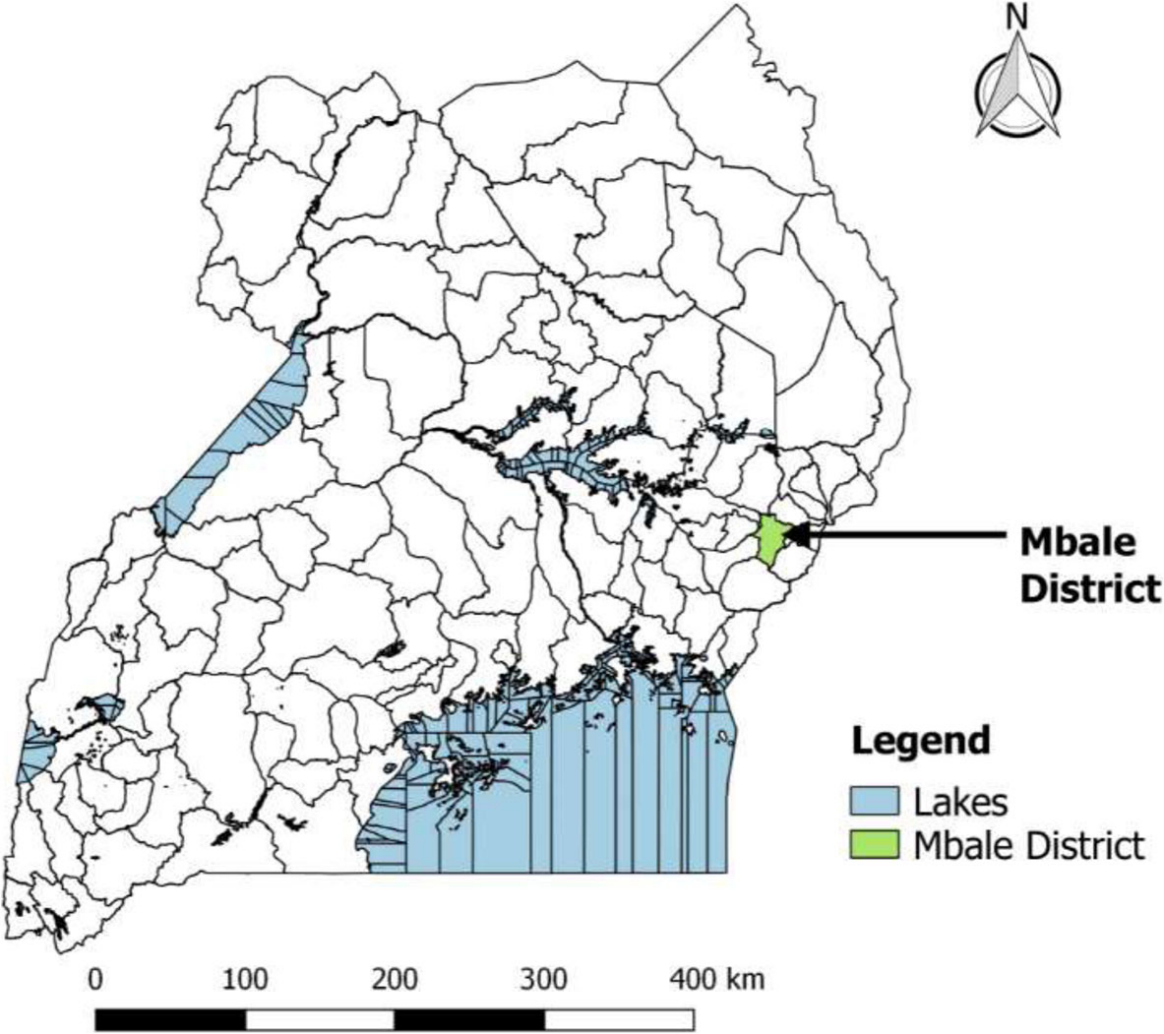
Map of Uganda showing location of Mbale District (shaded) in Eastern Uganda

The study was conducted in Mbale District in the Eastern Region of Uganda (Fig. 1). It was selected for this study because for the 5 years preceding the dates of this study, it recorded the highest number of animal bite cases in humans in Eastern Uganda [15]. The study included human and animal health professionals in the District health and production departments respectively. The former had 46 functional health facilities ranging from the lowest (Health Centre II) to the highest (Regional Referral Hospital). This included both government and privately owned health facilities. The health facilities were distributed as follows: 17 Health Centre II at parish level; 22 Health Centre III at subcounty level; 3 Health Centre IV at county level, 3 General Hospitals at district level and 1 Referral Hospital at regional level. According to Mbale District records, there were 261 workers in the health sector and 25 animal health workers.

### Study design and data collection

This was a cross sectional study conducted between December, 2012 and March, 2013. A sampling frame of human health professionals and animal health professionals was obtained from the district health and district veterinary offices respectively. Both qualitative and quantitative data collection methods were employed. Quantitative data was collected using pre-tested questionnaires administered to animal and human health professionals through face-to-face interviews. The questions covered knowledge, attitude and practices with respect to rabies management. Qualitative data was obtained from four (4) focus group discussions (FGDs) and 2 Key Informant (KI) interviews. The FGDs and KIs were administered in addition to the questionnaires for purposes of triangulation of data.

### Participant enrolment and sample size determination

The following inclusion criteria were set for the two categories of professionals: (a) An animal health worker present and working in Mbale District at the time of the study in the selected veterinary work station (subcounty), had formal training in animal health and had attained vocational college diploma or university degree and who consented to participate in the study by signing the consent form. (b) A human health worker qualified as a nurse, clinical officer or medical officer, present and working in Mbale District at the time of the study in the selected health facility and who consented to participate in the study by signing the consent form. Incomplete response to the questionnaire would automatically exclude a respondent from the study.

To obtain the sample size for animal health professionals and human health professionals, proportion-to-size sampling technique was used. This was followed by simple random sampling to obtain the respondents. Since the available records showed that there were 210 human health professionals (8 medical doctors, 4 clinical officers and 157 nurses) and 25 animal health professionals (9 veterinary officers and 16 animal husbandry officers), the study population considered in the sample size determination was therefore 235. For populations that are large, Cochran developed an equation to yield a representative sample for proportions [17]. That is,

$$ {n}_0=\frac{Z^2 pq}{e^2} $$

which is valid where n_0_ is the sample size, Z^2^ is the abscissa of the normal curve that cuts off an area α at the tails (1 - α equals the desired confidence level, for instance 95%), is the desired level of precision, *p* is the estimated proportion of an attribute (knowledge, attitude and practice) that is present in the population, and *q* is 1-*p*. The value for *Z* is found in statistical tables which contain the area under the normal curve [17]. Substitute for *Z* = 1.96, *p* = 0.5, *q* = 0.5, *e* = 0.05 in the above formula, *n*_*0*_ = 385. (p = 0.5 was used because the proportion of animal and human health workers who were knowledgeable about rabies in Mbale District was unknown. So, a 50 % proportion was assumed).

If the population is small then the sample size can be reduced slightly. This is because a given sample size provides proportionately more information for a small population than for a large population. The sample size (*n*_*0*_) can be adjusted using the following equation [17]:

$$ n=\frac{n_0}{1+\frac{\left({n}_0-1\right)}{N}} $$

Where *n* is the sample size and *N* is the population size. Therefore substituting for *n*_*0*_ = 385, *N* = 235 in above formula gives us, *n* = 146.16167 which approximates to 147. Therefore sample size estimation for both animal and human health workers was 147. Using proportion-to-size sampling, the number of human health professionals sampled was (210/235*147) = 131.361, which was approximately 131. The number of medical officers, clinical officers and nurses sampled were (8/210*131), (45/210*131) and (157/210*131) respectively which translates to 5 medical doctors, 28 clinical officers (graduates of a three-year diploma training in clinical medicine) and 98 nurses.

**Fig. 2:**
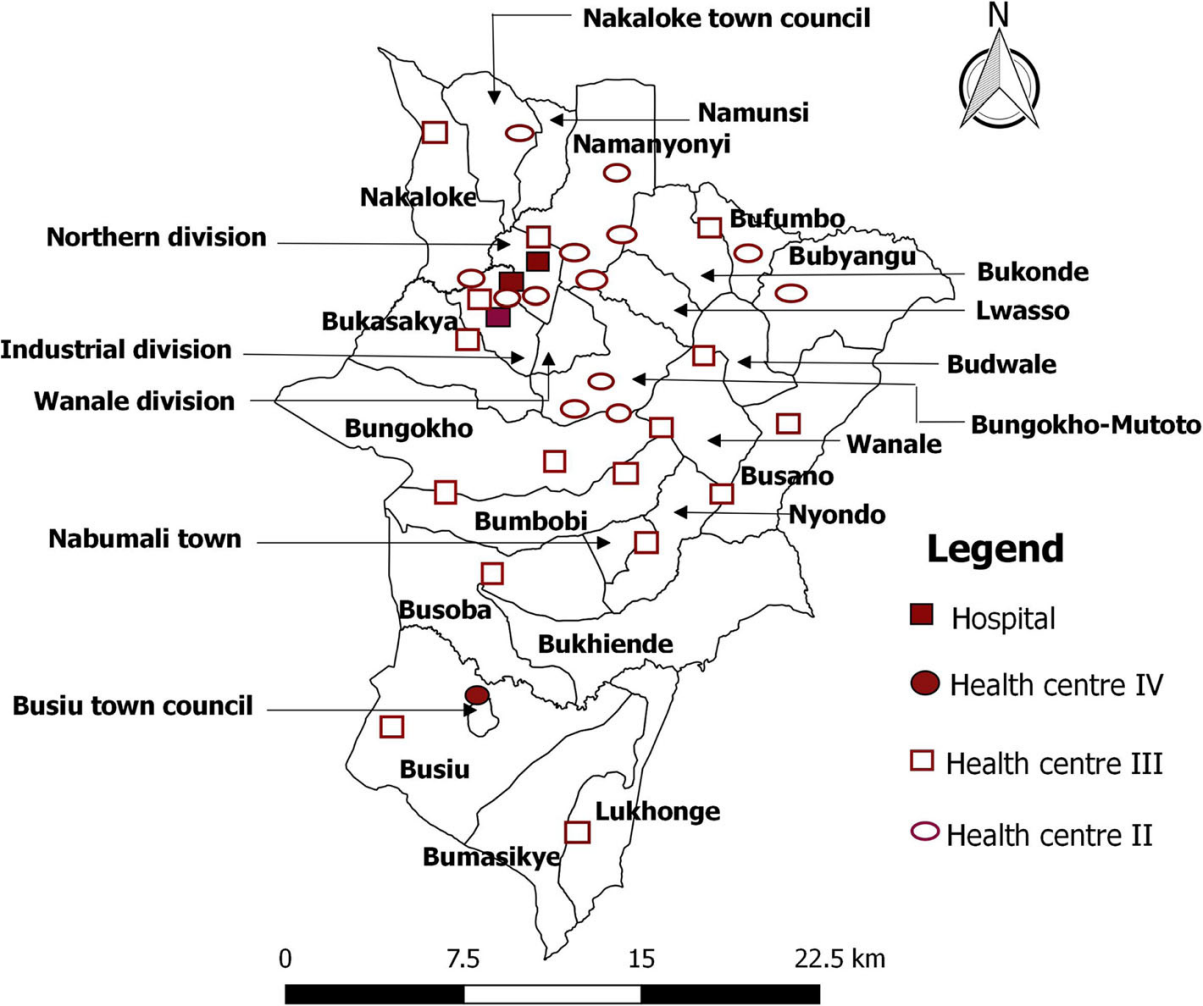
Distribution of health facilities sampled in Mbale District

Through a random selection process of health workers, a health facility to which the health worker belonged was automatically selected and subsequently visited. For purposes of being representative, the sampling process was done for each category of health worker and a tally done to ensure that at least 50% of the total number of health facilities and that all categories of health facilities were included. Consequently, a total of 33 health facilities were selected (Fig. 2).

Similarly, the total number of animal health professionals sampled was arrived at using proportion-to-size sampling (25/235*147 = 16). Proportionately therefore, 5 and 11 veterinary officers (degree holders) and animal husbandry officers (diploma holders) respectively were randomly sampled for this study. All selected respondents consented and participated in the study satisfactorily.

### Socio-demographic information

The following socio-demographic information was obtained from the respondents: their work department, sex, age, residence (rural or urban), religion, level of education, years spent in service, whether they owned dogs and/or cats or not. Dogs and cats were relevant in this study because they are the main culprits in animal bites and rabies transmission [6, 18].

### Measurement of knowledge

A method described by Koruk and others [19] was used to measure knowledge about rabies using 16 questions. The method was designed to measure knowledge among health professionals regarding rabies control hence its adoption for use in the Mbale study. A holistic approach to rabies control was important in this study. The questions included about rabies were: knowledge of the disease; its cause; main reservoirs; species affected; mode of transmission; groups of people most prone; incubation period in animals; signs/symptoms in animals; period of communicability in dogs/cats; incubation period in humans; signs/symptoms in humans; preventive measures; first aid given to a patient bitten/scratched by suspected rabid animal; prevention of rabies after an animal bite; vaccination regimen / schedule for pets; and vaccination regimen / schedule for humans (Additional file 1). Although not backed by a study, in Uganda government recommends annual vaccination of pets with a killed rabies vaccine. All these questions were equally weighted. Thus, a positive value of one (1) was assigned to each correct response, and a value of zero (0) was assigned to each wrong response or partially correct response. The average score for the respondents was then established. The scale was converted into a binary outcome basing on the average score. Respondents who scored above average mark were considered to have sufficient knowledge and those below average mark to have insufficient knowledge.

### Measurement of attitude

Attitude was measured on an ordinal scale using a three-point Likert scale (2 = agree, 1 = not sure, 0 = disagree) using ten statements. In this study, attitude referred to a person’s opinion or thoughts or viewpoint towards a given situation or scenario. The following statements were used in this study: think that rabies is not caused by bacteria; think that rabies affects warm-blooded animals; all wild and domestic animals are not the only source of rabies infection; believe that bats transmit rabies; knows that rabies can be transmitted through aerosols; would advise a person to seek treatment from a health facility / veterinary facility; willingness of the communities to vaccinate their pets; do you think vaccination of pets greatly contributes to rabies control in Mbale District; sensitization efforts against rabies would lead to rabies control; necessary to have joint efforts by the medical and veterinary sectors to control rabies (Additional file 1). The responses were collapsed into a binary outcome using a cut-off point of 86% as suggested by Dhand and others [19]. Those who agreed to 86% of the questions and below (less than 9 questions) were considered to have negative attitude. Those who agreed to more than 86% of the questions (9 or 10 questions) were considered to have positive attitude.

### Measurement of practices

Questions used to measure good or bad practices against rabies belonged to the following broad categories: roles played in anti- rabies campaign and how often; management of stray dogs; management or treatment of a patient bitten / scratched by a suspected rabid animal (Additional file 1). If a given practice was in conformity with the existing literature about prevention and control of rabies, then it was regarded as “good practice”. However, if the practice was contrary to the existing literature about prevention and control of rabies, then it was categorised as “bad practice”.

### Qualitative data collection

A total of four focus group discussions (FGDs) and two Key Informant (KI) interviews were conducted using an FGD and KI interview guides respectively. One FGD was conducted for clinical officers, two for nurses and one for veterinary staff. The two KIs were separately administered to a District Veterinary Officer and a Senior Public Health Officer. During both the FGD and KI, text notes were taken as the interviews (discussions) progressed.

### Statistical analysis

Qualitative data collected from FGDs and KIs was recorded in form of text notes, then analysed and summarised into themes, sub-themes and narratives. All questionnaire data was cleaned and entered into EpiInfo version 3.5.1, double-checked and exported to STATA (version 11.0) for further analysis. The descriptive statistics generated for discrete variables were absolute frequencies (raw counts), and relative frequencies (proportions or percentages of total number of observations). The descriptive statistics generated for continuous variables were mean and standard deviation. Thus, the key outcomes (knowledge, attitude and practices) were expressed as proportions. Age of respondents and years in service were categorised. Age ranges used were: ≤29, 30–49, 50–69 [first third, second third and last third of life – the average life expectancy in Uganda is 50.4 years] [20], while years in service were categorised as 1–20 and 21–40 (first and second halves of formal working life).

### Bivariate data analysis

#### Knowledge

Since the outcome of interest was binary (sufficient knowledge or insufficient knowledge), a Chi-square test was applied. If in any one of the cells, the expected observation was less than 5, then a Fisher’s exact test was used. The Chi-square test or Fisher’s exact test assessed the association between knowledge and independent variables such as education level. In addition, the Chi-square test or Fisher’s exact test assessed the association between knowledge and attitudes. Also, the relationship between knowledge and practices was established. If the *p*-value generated was < 0.05, then the association was considered significant.

#### Attitudes

The outcome of interest being binary (positive attitude or negative attitude), a Chi-square test was used. If in one of the cells, the expected observation was less than 5, then a Fisher’s exact test was employed. The Chi-square test or Fisher’s exact test assessed the association between attitudes and practices. If the *p*-value generated was < 0.05, then the association was considered significant.

#### Practices

Both good and bad practices were established based on the factual basis of responses.

### Multivariate data analysis

To control for confounding among the associated factors, multivariate analysis was adopted. The following criteria were used in selecting variables into multivariate analysis – that is binary logistic regression model: 1) variables which were significant (*p* < 0.05) at bivariate analysis; 2) variables which had a *p*-value above 0.05 but less than 0.2; and 3) scientific plausibility of the variables.

#### Knowledge

To establish factors affecting knowledge of rabies, all the factors that met the above criteria were entered into binary logistic model. Using backward stepwise elimination, the factors were dropped one by one until the best model was obtained. The best model was determined using the Hosmer and Lemeshow goodness of fit test [20, 21]. If the p-value generated by the goodness of fit test was greater than 0.05, it would suggest that the model fitted the data well and that the factors obtained were significant predictors of level of knowledge of rabies reported in the study.

### Ethical issues

Approval was sought from Makerere University College of Veterinary Medicine, Animal Resources, and Bio-security (COVAB) Higher Degrees and Research Committee. Permission was also sought from Mbale District Local Government Authorities and Mbale Regional Referral Hospital. Written consent to participate in the study was sought from respondents on an individual basis.

## Results

### Socio-demographic characteristics of study participants

A total of 147 respondents participated in the study: 5 medical officers, 28 clinical officers; 98 nurses, 5 veterinarians and 11 animal husbandry officers. The average age of the respondents was 35.07 ± 9.4 years as shown in Table 1. There were more female (56%) than male (44%) respondents. Most respondents lived in rural areas (71%) compared to urban (29%). More respondents identified as Christians (90%) compared to Muslims (10%). There were more married (80%) than single (20%) respondents; less degree holders (7%) than diploma (40%) and certificate (52%) holders. Fewer respondents had served for more than 21 years (12%) compared to those who served for 20 years and less (88%). The respondents who did not own dogs and cats were more (84 and 92% respectively) than those who owned (16 and 8% respectively). Dogs and cats were relevant in this study because they are the main culprits in animal bites and rabies transmission.

**Table 1:** Socio-demographic characteristics of animal and human health workers

	Socio-demographic characteristics	Animal Health Professionals, ***n*** = 16 (%)	Human Health Professionals, ***n*** = 131 (%)	Overall, ***n*** = 147 (%)
**Location**	Urban	0	43 (33)	43 (29)
	Rural	16 (100)	88 (67)	**104 (71)**
**Sex of respondent**	Male	14 (88)	50 (38)	64 (44)
	Female	2 (12)	81 (62)	**83 (56)**
**Marital status**	Single	7 (44)	111 (85)	29 (20)
	Married	9 (56)	20 (15)	**118 (80)**
**Age group (years)**	≤29	10 (63)	41 (31)	51 (35)
	30–49	5 (31)	74 (56)	**79 (54)**
	50–69	1 (6)	16 (13)	17 (12)
Mean age (SD)		32.88 (± 9.44)	35.34 (± 9.39)	35.07 (± 9.40)
**Qualification**	Certificate	0	77 (59)	**77 (52)**
	Diploma	11 (69)	48 (37)	59 (40)
	Degree	5 (31)	6 (5)	11 (8)
**Religion**	Christian	14 (88)	13 (10)	**132 (90)**
	Muslim	2 (13)	118 (90)	15 (10)
**Years in service**	1–20	15 (94)	115 (88)	**130 (88)**
	21–40	1 (6)	16 (12)	17 (12)
**Dog ownership**	Own dogs	5 (31)	18 (14)	23 (16)
	Don’t own dogs	11 (69)	113 (86)	**124 (84)**
**Cat ownership**	Own cats	1 (6)	11 (8)	12 (8)
	Don’t own cats	15 (94)	120 (92)	**135 (92)**

Bold values: Highest percentage in the respective categories

### Knowledge about rabies

Knowledge about rabies was gauged based on the scores by the respondents (Table 2). The cut-off for sufficient knowledge was adopted from Koruk and others (2011). The average score for both categories of professionals (cut-off) in this study was 8.3 marks (Table 2). Respondents who scored over 8.3 were considered to have sufficient knowledge and those who scored below 8.3 were regarded as having insufficient knowledge. Findings showed that less than half of all the respondents (44%, 65/147) and less than half of human health professionals (41%, 53/131) had sufficient knowledge about rabies. On the other hand, three quarters of the animal health professionals (75%, 12/16) had sufficient knowledge about rabies (Table 2).

**Table 2:** Knowledge scores attained by respondents about rabies

Knowledge scores attained	Number of Animal Health Professionals, ***n*** = 16 (%)	Number of Animal Health Professionals, ***n*** = 131 (%)	Overall, ***n*** = 147 (%)	Total number of scores for both (product of scores attained and number of professionals)
**2**	0	1 (1)	1 (1)	2
3	0	4 (3)	4 (3)	12
4	0	7 (5)	7 (5)	28
5	0	10 (8)	10 (7)	50
6	1 (6)	15 (11)	16 (11)	96
7	1 (6)	19 (15)	20 (14)	140
8	2 (13)	22 (17)	24 (16)	192
9	1 (6)	17 (13)	18 (12)	162
10	4 (25)	11 (8)	15 (10)	150
11	2 (13)	9 (7)	11 (7)	121
12	1 (6)	9 (7)	10 (7)	120
13	2 (13)	4 (3)	6 (4)	78
14	1 (6)	2 (2)	3 (2)	42
**15**	1 (6)	1 (1)	2 (1)	30
				Average = 8.3

### Knowledge about source of infection and transmission modes

The majority of respondents (69%, 102/147) knew that the reservoirs of rabies were “wild and domestic canids and other biting mammals”. The majority of animal health workers (81%, 13/16) knew about the reservoirs of rabies compared to 68% (89/131) human health workers.

Almost all the respondents (97%, 143/147) knew that rabies was transmitted through bites or scratches from rabid animals (all the 16 animal health and 127 human health workers). More than a half (51%, 75/147) of the respondents reported children to be more prone to animal bites than any other category of people.

Similar findings were revealed in FGDs and KIs. For instance a participant said: *“Dogs, cats and jackals are the main species that carry and spread rabies”* (FGD, clinical officers). Likewise, in another FGD, a participant said: “*Rabies is transmitted through infected saliva from bites of infected animals or scratches”* (FGD 1, nurses).

In yet another FGD, a participant stated:

“*Since my high school, I know that rabies is spread through a bite or scratch from infected dogs or cats, so as much as possible I always avoid provoking dogs/cats. When I find a dog alone on the way, I avoid passing near this dog”* (FGD, clinical officers).

In one KI, a respondent said: “*Jackals, canines and felines are the main reservoirs here in Mbale District”* (KI, Veterinary Department). In another KI, a respondent said: “*Rabies is transmitted through animal bites and scratches”* (KI, veterinary staff).

However, in one of the FGD’s, a participant said: “*all people are bitten by dogs equally”* (FGD, veterinary staff).

Another participant said:

“In *my village, dogs and foxes are the only animals that spread rabies by biting people. I have never seen a cat with rabies chasing people to bite them. If these mad dogs or foxes bite people, these people also run mad after some time”* (FGD 2, nurses).

### Knowledge on control of rabies

The majority (92%, 135/147) of respondents knew that rabies is prevented by vaccination of pets against rabies in general. Likewise, the majority (80%, 118/147) knew that after a suspected animal bite, rabies could be prevented by taking the patient to health centre for administration of post exposure prophylactic treatment. About two-thirds (65%, 96/147) of the respondents knew that the vaccination schedule against rabies for pets was once a year.

In one of the FGDs, a participant said: “*Rabies can be prevented through vaccination of dog/cats against rabies and mass sensitization of communities against rabies”* (FGD, veterinary staff). In another FGD, a participant said: “*If we are serious and want to control rabies then we should always immunize our pets against rabies, sensitize our societies about dangers of rabies and report cases of dog bites to a health centre”* (FGD 2, nurses). Also in a Key informant Interview, a respondent said:

*“We carry out sensitizations through radios, trainings, and meetings. However, no funds to do big anti-rabies campaign (i.e. transport/fuel). There is a small vote under marketing and grant (some form of disease control) - about 5million shillings annually. It comes on a quarterly basis and not enough. Otherwise, the veterinarians in the field do it on their own”* (KI, Veterinary Department).

### Factors influencing knowledge of rabies at multivariate analysis

Table 3 shows the factors from bivariate analysis that fulfilled the criteria for use in the building of the multivariate logistic regression model. The only factors that were significant (*p* < 0.05) in the model were refresher trainings (OR: 4.89; 95% CI: 1.70–14.09) and qualification (OR: 3.16; 95% CI: 1.17–8.53). Thus, having refresher trainings and the type of qualification were the two factors significantly associated with the level of knowledge about rabies. A nurse had 0.12 times lower odds of being knowledgeable about rabies (OR: 0.12, 95% CI: 0.01–1.20) than any other category of profession in the study. Respondents with a degree qualification had almost 13 times higher odds (OR: 12.83, 95% CI: 2.19–74.99) of being knowledgeable about rabies than those with either a diploma or certificate. Respondents who had attended a refresher training had a 6 times higher odds (OR: 6.17, 95% CI: 2.39–15.90) of being knowledgeable about rabies than those who had not.

**Table 3:** Factors influencing level of knowledge about rabies

Factors	OR(95% C.I)	***P***-value
Location	0.74 (0.30--1.81)	0.50
Department	0.34 (0.01--10.76)	0.542
Gender	1.34 (0.56--3.24)	0.51
Designation	0.99 (0.30--3.24)	0.98
Qualification type	3.16 (1.17--8.53)	**0.02**
Tribe	0.10 (0.86--1.16)	0.97
Refresher trainings	4.89 (1.70--14.09)	**0.00**
Collaborative effort among staff	0.57 (0.09--3.65)	0.55

Key: Bold *p*-values = significant *p*-values

### Testing of the binary logistic model for the level of knowledge about rabies

The most recommended test for overall fit of a binary logistic regression model is the Hosmer and Lemeshow test (H-L test). If the H-L goodness of fit test statistic for the level of knowledge about rabies is greater than 0.05, then the model adequately fits the data. When the H-L goodness of fit test was done, it yielded a *p*-value of 0.57. The p-value generated being greater than 0.05, suggested that the model fitted the data well and that the two factors (refresher trainings and type of qualification) were significant predictors of the level of knowledge of rabies reported in the study.

### Attitude towards rabies management

A quarter of the respondents (25%, 37/147) had positive attitude towards rabies management. Furthermore, less than half of animal health workers (44%, *n* = 7/16) and less than a quarter of human health workers (23%, *n* = 30/131) had positive attitude towards rabies management. While level of education influenced attitude towards rabies, years in service did not. Generally, respondents who had degrees had 7 times higher odds (OR = 7.23, 95% CI: 1.71--30.66) of having positive attitudes than those with lower qualifications (diplomas or certificates). There was no significant difference within professions.

### Practices towards rabies management

Half of the respondents (50%, 74/147) knew good practices towards rabies management. They were knowledgeable about the following good practices: vaccination of pets (5%, 8/147) respondents, vaccination of humans (10%, 14/147), community sensitization (27%, 40/147), sensitizing people when approached (4%, 6/147) and vaccination of pets and sensitizations (4%, 6/147). The major bad practice demonstrated by the majority of respondents (73/147) was “not participating in any anti-rabies campaign”.

### Association between level of knowledge, attitude and practices towards rabies management

Statistical analysis revealed a significant association between attitude towards rabies management and level of knowledge about rabies (*p* value = 0.00). A respondent with sufficient knowledge about rabies had a 3.65 times higher odds of having a positive attitude towards rabies management (OR = 3.65; 95% CI: 1.60–8.31) than one with insufficient knowledge. There was also a significant association between attitude towards rabies management and practices towards rabies management (p value = 0.04). A respondent with a positive attitude towards rabies management had 2.22 times more higher odds of having good practices against rabies (OR: 2.22; 95% CI: 1.01–4.86) than a respondent with a negative attitude. In contrast, there was no significant association between level of knowledge of rabies and practices towards rabies management by respondents (*p* value = 0.16).

## Discussion

Guided by the fact that rabies is a zoonotic disease, our respondents came from both the veterinary department and human health department of Mbale district where the key stakeholders involved in rabies control belong. This study found that less than half of all the respondents interviewed had sufficient knowledge about rabies though there were differences among professions. The majority (60%) of human health professionals interviewed had insufficient knowledge about rabies. Similar findings were reported in a Turkish study which found that physicians had insufficient basic and clinical knowledge of rabies [19, 21]. A related study in India pointed out that there were numerous gaps in the knowledge of doctors regarding animal bite management [22] just like in Pakistan where General Practitioners were found to have poor knowledge regarding dog bite management [23]. Additionally, in India, a study by Chowdhury and others [24] revealed that there were significant gaps in knowledge regarding the management of animal bite injuries and immunization among interns of a government medical college in Kolkata. In a more general study in Tanzania, Swai and others [11] reported that staff in human health facilities had a low awareness and poor knowledge of zoonoses. This study, also found out that the level of knowledge of rabies influenced attitude which in turn influenced practices towards rabies management. This is in agreement with the findings of Mascie-Taylor and others [25], which showed that the level of knowledge influences attitudes and practices. It must also be noted that our study demonstrated that the level of training (educational qualification) and the period of service (experience) play a significant positive role in the level of knowledge about rabies. Our findings therefore suggest that there is need to enhance knowledge about rabies among health workers so as to influence their attitude and practices against rabies.

In contrast to human health professionals, majority (75%) of the animal health professionals had sufficient knowledge about rabies. This finding contradicts that from a study in neighbouring Tanzania which indicated that animal health workers or practitioners had poor knowledge about zoonoses (rabies inclusive) [11]. Swai and others further pointed out that knowledge levels, specifically about rabies as zoonosis in comparison with other zoonoses, was deemed to be good among animal health staff or practitioners. The difference in findings of the two studies could probably be explained by the fact our study focused specifically on rabies while that of Swai and others was on zoonoses in general [11].

Only 25% of all the respondents agreed to at least 9 statements about attitude towards rabies management and hence had positive attitudes. This implies that the majority of them had negative attitude towards rabies management. Similar proportions were also found among respondents from different professions (human and animal health workers) with minor variations. This negative attitude towards rabies management was in agreement with related studies done in Kenya [26]. The latter study found negative attitudes among respondents and recommended that public health workers needed more knowledge, correct attitude and skills to enable them conduct surveillance and teach public control measures against zoonotic diseases.

This study revealed that half of the overall respondents were not involved in any of the known anti-rabies control campaigns. This was probably due to inadequate emphasis on anti-rabies campaigns in Mbale District. The latter ought to borrow an approach used by its neighbour, Bududa District, where in April 2009 the District Local Government, the Ministry of Agriculture, Animal Industry and Fisheries, Uganda Veterinary Association, Makerere University Veterinary School and other partners joined efforts to carryout a district-wide rabies vaccination and community sensitisation campaign [27].

Additionally, less than half of the respondents were involved in good practices aimed at reducing stray dog population in Mbale District. This is possibly due to the inability of animal owners to afford the costs of spay and neuter which are the major methods of dog population control in Uganda. Furthermore, less than half of all respondents were involved in good practices regarding management of a person bitten by a suspected rabid dog.

Okech and others [28], argue that the current good practice in rabies control is to embrace the “One Health” approach in which animal health and human health professionals and other key stakeholders work together in community awareness and animal vaccination campaigns. One Health recognises that the health of humans, animals and the environment are interconnected. One Health focuses on collaborative efforts that harness and coordinate the power of multidisciplinary and cross-sectoral teams and resources so as to apply them locally, nationally and internationally for optimum health of humans, animals and the environment [29]. The common theme in application of One Health approach in management of rabies is collaboration across disciplines and sectors.

Although this study involved only professionals in Mbale District and not the entire eastern region or country, its findings could still be useful in guiding intervention strategies in other parts of the country. This is especially so because rabies is endemic in every part of the country, all districts draw their professionals from the same pool of training institutions and the districts have similar resource challenges.

The revelation, by this study, of associations between attitude and knowledge, attitude and practice, and knowledge and practice informs the design of intervention programmes. A sufficient focus on empowering professionals with knowledge through upgrading their qualifications and conducting regular refresher training is one of the many approaches that ought to be considered in rabies management and control programmes.

## Conclusion and recommendations

Unlike their animal health professional counterparts, the human health professionals in Mbale District had insufficient knowledge about rabies. Both animal and human health professionals in Mbale Distric had a negative attitude towards rabies management. There were also limited good practices in rabies management by both animal and human health professionals. This calls for regular refresher trainings for animal and human health professionals about rabies to improve their knowledge and attitudes towards its management. This would in turn have a positive effect in improving practices towards rabies control. The district also ought to consider adopting the “One Health” approach to rabies control. This approach would entail formation of multi-sectoral, multi-disciplinary (veterinarians, medical professionals, wildlife specialists, environmental public health experts, social workers) district teams that would complement each other in their rabies control efforts. This would entail working and learning together and from each other, joint community sensitisation and joint reporting/sharing of information. Such an initiative calls for harnessing of resources from various sources and focusing them to achieve a common goal.

## Supplementary Information

**Additional file 1.**

## Data Availability

The datasets during and/or analysed during the current study available from the corresponding author on reasonable request.

## Abbreviations

CI: Confidence Interval
CoVAB: College of Veterinary Medicine, Animal Resources and Biosecurity
FGD: Focus Group Discussion
HMIS: Health Management Information Systems
KAP: Knowledge, Attitude and Practices
KI: Key Informant
OR: Odds Ratio
*P*-value: Probability value
SD: Standard Deviation
&: And
<: Less than
>: Greater than
