# Supplementary material for: Knowledge, attitude and practices about rabies management among human and animal health professionals in Mbale District, Uganda
Source: One Health Outlook. 2020 Dec 14;2:24. doi: 10.1186/s42522-020-00031-6 (PMC7993504; doi:10.1186/s42522-020-00031-6)
Supplement: Supplementary file 1 — Additional file 1. [file 42522_2020_31_MOESM1_ESM.docx]

**Appendix 1**

**Questionnaire for KAPS**

**To establish knowledge, attitudes and practices regarding rabies management among animal and human health workers in Mbale District, Uganda**

Background and consent

My name is Fred Monje a student of Makerere University, college of veterinary medicine, animal resources, and bio-security (COVAB). We are interviewing animal and human health workers working in Mbale District about rabies. This will help us generate practical and yet cost effective control measures to the problem of dog bites and rabies in the district. Some questions may be somewhat sensitive but they are important for the success of this study. Specifically, we would like to learn about your personal knowledge, attitudes and practices towards rabies management in Mbale District. We would like also to inform you that there is no risk to participation in the study. Participation in this study is voluntary. Everything that you share with me will be kept confidential. We therefore need your assistance. You may ask me to clarify questions if you do not understand them. I will not write your name down anywhere on the questionnaire, and no one will be able to link your answers to your identity.

Do you agree to participate in this study?

a) yes

b) no

Signature________________________

Thank you, we can now start.

Questionnaire number………. …. Subcounty………………Parish……………Village…………………

| Name of your department | | 1) veterinary | | 2) health |
| --- | --- | --- | --- | --- |
|  | **A. Socio-demographic characteristics** | | |  |
|  | Instruction: Please tick as appropriate | |  |  |
|  | **1.** What is the gender | of the respondent? 1) male 2) female | | |

1. a) How old is the respondent? (Answer in years)……… …………………

b)Marital status of the respondent. 1) single 2) married 3)separated/divorced/widowed

1. Which year were you born?.........................................................
2. How many years have you been in service?……………………
3. What is the name of your present place of work?……………………………
4. What is the designation of respondent? 1) medical officer 2) clinical officer 3) nurse 4)veterinary officer 5) animal husbandry officer 6) others, specify…………………….
5. What is your highest level of Education attained? 1) Secondary 2) tertiary -university 3)tertiary-

non university 4) others, specify………………....

1. What qualification did you attain? (Specify qualification)……………………………
2. What is the religion of respondent? 1) christian 2) moslem 3) others, (specify)………
3. What is your tribe? ………………………………

28

**11.** a)Do you own any domestic animal? 1) yes 2) no

b) If yes, state their number. 1) cattle……….2) goats… 3) sheep….. 4) pigs 5) dogs….. 6)

cats………….. 7) Others, (specify)………

**B. Knowledge of rabies**

Instruction: Please tick the answer given by the respondent. More than one option is possible here.

1. **Knowledge of causes of rabies**
2. Do you know about rabies? 1) yes 2) no 3) not sure
3. If yes what do you know about rabies? 1) Fatal disease 2) is zoonotic 3) only in Mbale 4) worldwide 5)viral 6) bacterial 7) others, (specify)…………

**3.** What causes rabies? 1) Bacteria 2) virus 3) protozoa 4) fungi 5) others, (specify)………

**ii)** **Knowledge on source of infection and transmission modes**

1. What are the main reservoir(s) of rabies in Mbale District? 1) Many wild and domestic canidae and

other biting mammals 2) only wild canidae 3) only domestic canidae 4) all animals 5) others,(specify)……….

**5.** What species does rabies affect? 1) Animals 2) humans 3) all mammals 4) dogs 5) cats 6) others, (specify)………………………………….

1. How is rabies transmitted? 1) bite or scratch of infected animal 2) witch craft 3) intact mucous membranes 4) do not know 5) others, (specify)……………………..
2. What groups of people are most prone to animal bites? 1) Adult people 2) children 3) youth 4) Old people 5) do not know 6) others, (specify)………………………

**iii) Knowledge on manifestation and control of rabies**

1. What is the incubation period of rabies in animals? 1) 10days to 2 or more months 2) 25days to 150 days or more 3) 1 to 3days 4) do not know 5) others, (specify)…........

| **9.** | List the signs of rabies in animals? 1) change in behavior, hide in dark corners | | 2)aggressive with a |
| --- | --- | --- | --- |
|  | tendency to | bite moving objects 3) profuse salivation 4) convulsions 5) | paralysis 6) others, |
|  | (specify)………………………………… | |  |
| **10.** | What is the period of communicability of rabies in dogs/cats? 1) one month | | 2) 3-7days 3) one |
|  | year 4) 7years 5) do not know 6) others, (specify)…………………………. | |  |
| **11.** | What is the incubation period of rabies in humans? 1)3-4days 2) 3-8weeks 3) 9days to 7years 4) | | |
|  | do not know | 5) others, (specify)……………………………. |  |

1. List the signs of rabies in humans. 1) paresis or paralysis 2) delirium 3) convulsions 4) without

medical attention, death in about 6days 5) do not know 6) others, (specify)…………………

1. How do we prevent rabies? 1) vaccination of pets against rabies 2)sensitization of communities against rabies 3) apply active surveillance for rabies in animals 4)detain and clinically observe for

10 days any healthy appearing dog or cat known to have bitten a person 5) submit immediately to a

lab the intact heads packed in ice of animals that die of suspected rabies 6)euthanize immediately non vaccinated dogs or cats bitten by known rabid animal 7) do not know 8) others, (Specify)………………..

29

1. What first aid is given to a patient after a bite/scratch from a suspected rabid animal? 1) immediate and thorough cleaning of the wound with soap, followed by ethanol or iodine 2) suture the wound
   1. apply some herbs 4) do not know
      1. Others, (specify)………………….
2. How do you prevent rabies after an animal bite? 1) immediate and thorough cleaning of the wound with soap or detergent and water 2) take to health centre for administration of human rabies immune globulin as soon as possible 3) suture the wound 4) do not know 5) others, (specify)……………………...
3. What is the vaccination regimen/schedule for pets against Rabies? 1) once a year 2) once in a life

time 3) once every 2years 4) do not know 5) others, (specify)……….

1. What is the vaccination regimen/schedule for humans against Rabies? 1) once a year 2) vaccinate

high risk groups 3) once a life time 4) do not know

5) others, (specify)……………….

1. **Attitudes**

Instruction: please tick the answer which is applicable to you

1. Believe that rabies is not caused by bacteria. 1) Agree 2) not sure 3) disagree
2. Think that rabies affects warm blooded animals. 1) agree 2) not sure 3) disagree

| **3.** | All wild and domestic animals are not the |
| --- | --- |
|  | only source of rabies infection. 1) agree 2)not sure 3) disagree |

1. Believe that Bats transmit rabies. 1) agree 2) not sure 3) disagree

**5.** Knows that rabies can be transmitted through aerosols. 1) agree 2) not sure 3)disagree

1. Would advise a person bitten/scratched by suspected rabid animal to seek treatment from a health facility /veterinary facility 1) agree 2) not sure 3)disagree
2. Communities are willing to vaccinate their pets. 1) agree 2) not sure 3) disagree
3. Thinks that vaccination of pets greatly contributes to rabies control in Mbale District? 1 ) agree 2)not sure 3)disagree

**9**. Sensitization efforts would lead to rabies control in Mbale District. 1) agree2) not sure 3) disagree

**10**. Necessary to have joint efforts by the medical and veterinary sectors to control rabies. 1) agree 2) not sure 3) disagree

**D. Practices**

Instruction: please tick the right answer or fill in the right answer as appropriate

**1.** Are you involved in any anti-rabies campaign in Mbale District 1) yes 2) no

1. What anti-rabies campaign are you involved in? 1) Vaccination in pets 2) vaccination in humans 3)

sensitization of communities against rabies 4) no 5) others, specify……………………

1. What role(s) do you play in anti- rabies campaign? ............................................

30

1. a) How often are you involved in anti- rabies campaign? 1) Once a week 2) once a month 3) once a year 4) others, specify…………………………………………..

b) Have you ever had a refresher training/workshop about rabies? 1)yes b) no

c) Have you ever had a joint collaborative effort by medical and veterinary departments to control rabies? 1) yes 2)no

1. How do you manage stray dogs/cats in Mbale District?..................................................
2. How do you treat a person bitten/scratched by a suspected rabid dog/cat in Mbale District…………………………
3. What are the challenges you encounter in managing a person bitten by a suspected rabid animal?
4. What are the challenges in rabies control in Mbale District?..........................
5. How should we overcome these challenges?....................

**End**

**Thank you!**

31
